# *Sec61β* Deficiency Disrupts *Drosophila* Oogenesis Through UPR-Mediated Defects in Germ Cell Proliferation and Differentiation

**DOI:** 10.3390/ijms27177640

**Published:** 2026-08-26

**Authors:** Zhi-Xian Cao, Xiao-Min Xiao, Yi-Ming Ji, Qian Wang, Chuan-Xiang Wu, Yan-Qiu Wang, Yu-Feng Wang

**Affiliations:** Key Laboratory of Pesticide & Chemical Biology of Ministry of Education, Hubei Key Laboratory of Genetic Regulation and Integrative Biology, School of Life Sciences, Central China Normal University, Wuhan 430079, China; caozxsweet722@163.com (Z.-X.C.); ccnuxxm@hotmail.com (X.-M.X.); 17371995820@163.com (Y.-M.J.); qianwang@mails.ccnu.edu.cn (Q.W.); 13668358907@163.com (C.-X.W.); wangyanqiu@gzxyyz.cn (Y.-Q.W.)

**Keywords:** *Drosophila melanogaster*, *Sec61β*, oogenesis, UPR, JAK/STAT, autophagy

## Abstract

*Sec61β* encodes one of the subunits of the Sec61 translocon, which is essential for translocation of the nascent polypeptides to the endoplasmic reticulum (ER). Knockdown of *Sec61β* in the testis leads to male sterility in *Drosophila melanogaster*. Although *Sec61β* is expressed in both testes and ovaries, its role in female reproduction remains unclear. In this study, we found that knockdown of *Sec61β* in either germ cells or somatic cells of ovaries causes complete female sterility, with a small size of ovaries from larval to adult stages, significant reduction in germ cell numbers beginning in the late third-instar larval stage and a complete absence of germ cells in pupal and adult ovaries. Overexpression of *Sec61β* efficiently rescues these defects. Furthermore, we observed that *Sec61β* knockdown in the germline triggers the unfolded-protein response (UPR), which not only non-autonomously activates JAK/STAT signaling in adjacent somatic cells to inhibit *Bam* expression but also autonomously impedes the G1/S phase progression of germ cells. These effects collectively impact cell proliferation and differentiation. Our results suggest that *Sec61β* is essential for germ cell maintenance via UPR-mediated cell-autonomous proliferation control and non-autonomous JAK/STAT signaling in *Drosophila* ovaries.

## 1. Introduction

Oogenesis depends upon basic cell biological processes including cell division, signal transduction, cell migration, and morphogenesis to create oocytes capable of fertilization. This variety of cell types and biological processes makes the *Drosophila* ovary a tremendous model for investigating an array of scientific questions. Oogenesis of *D. melanogaster* encompasses embryonic primitive gonad coalescence, larval ovary morphogenesis, pupal ovary formation and the 14 stages of adult follicle development [1].

At embryonic stage 17 (ES 17), two round gonads develop at the posterior pole of the embryo. Each gonad consists of approximately 12 primordial germ cells (PGCs) enveloped by somatic gonadal cells [2,3,4]. During the larval stages, the gonads undergo coordinated growth, with two major cell lineages, PGCs and somatic gonadal precursors, co-developing in a synergistic manner. PGCs proliferate to approximately 100 cells [5]. The somatic gonadal precursors undergo proliferation and differentiation to form distinct somatic cell types, including Terminal Filament Cells (TFCs), Cap Cells (CCs) situated adjacent to TFCs, and Intermingled Cells (ICs) enveloping PGCs. These cell types collectively constitute the ovarian niche [6]. In the late larval stage, PGCs associated with CCs differentiate into Germline Stem Cells (GSCs), which serve as the lifelong source of egg production in adult flies [7]. Meanwhile, at the late third-instar larval stage (LL3), 8–10 TFCs undergo flattening, sorting, intercalation, and stacking to form 16–20 aligned terminal filaments (TFs) [8,9]. TFs represent the initial sites of ovariole assembly and act as primary structural components of the ovarian niche [10,11].

**Figure 1:**
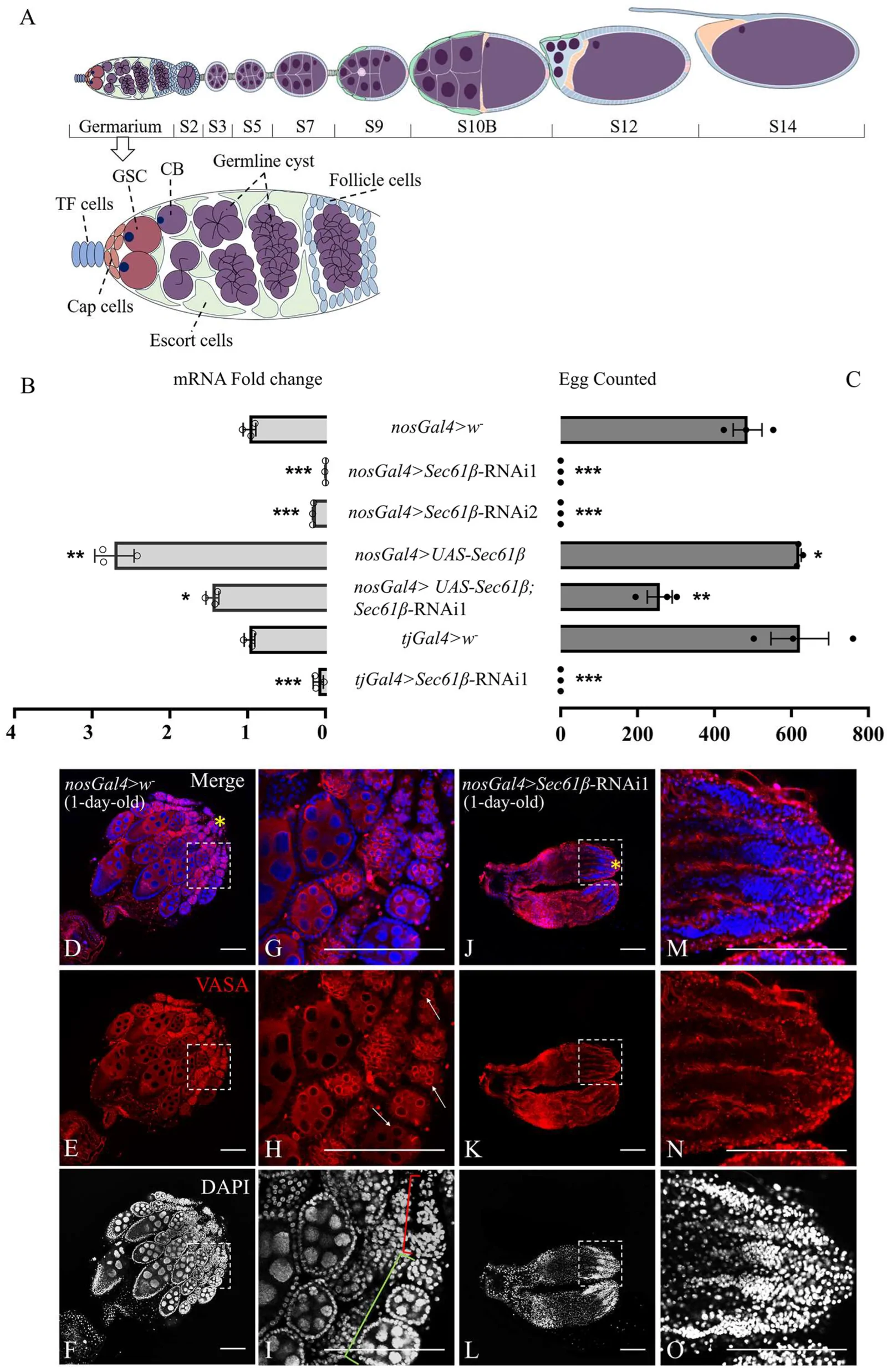
*Sec61β* is essential for female fertility in *Drosophila melanogaster*. (**A**) Schematic diagram of the different phases of *Drosophila* oogenesis. (**B**) qRT-PCR analysis of *Sec61β* expression levels in ovaries from *nosGal4* > *w*^−^ (control), *nosGal4* > *Sec61β*-RNAi1 (knockdown), *nosGal4* > *Sec61β*-RNAi2 (knockdown), *nosGal4* > UAS-*Sec61β* (overexpression), and *nosGal4* > UAS-*Sec61β*; *Sec61β*-RNAi1 (rescue) and *tjGal4* > *Sec61β*-RNAi1 (knockdown in somatic cell) flies. (**C**) The number of eggs laid by the corresponding females after crossing with wild-type male flies. The white dots in (**B**) and the black dots in (**C**) represent biological replicates. Data are presented as mean ± SE. * *p* < 0.05, ** *p* < 0.01, *** *p* < 0.001. (**D**–**I**) Control ovaries and (**J**–**O**) *Sec61β* knockdown ovaries from 1-day-old flies. (**D**–**F**) Overall appearance of control ovaries. (**J**–**L**) Overall appearance of *Sec61β* knockdown ovaries, showing a markedly smaller and atrophic morphology. (**G**–**I**) Anterior region of control ovarioles. The ovariole is composed of the germarium and egg chambers (red and green square brackets in (**I**)) with germ cells at various developmental stages (arrows in (**H**)). (**G**–**I**) are magnified views of the white dashed boxes in (**D**–**F**). (**M**–**O**) Anterior region of *Sec61β* knockdown ovarioles, displaying the germarium. The ovarioles lack germ cells. (**M**–**O**) are magnified views of the white dashed boxes in (**J**–**L**). Anti-VASA antibody (red) labels germ cells. DAPI (blue and white) labels cell nuclei. Asterisks indicate the germarium regions. Scale bars: 100 μm.

Around the larval–pupal transition, the ovaries lack organized ovarioles. By 12 h after puparium formation (APF), ovarioles become discernible [12], each consisting of an apical germarium and a posterior chain of developing egg chambers. GSCs reside in the niche at the germarium (Figure 1A). During oogenesis, GSCs divide asymmetrically to generate two daughter cells: the one in contact with CCs retains self-renewal capacity, while the distal daughter differentiates into a cystoblast (CB) [13]. Each CB undergoes four incomplete mitotic divisions, thereby generating 16 germ cells (GCs) containing one oocyte and 15 nurse cells, which collectively form a 16-cell cyst [13,14,15]. By 56 h APF, the first egg chamber, consisting of a 16-cell cyst surrounded by somatic follicle cells, becomes visible in ovarioles, and approximately 3–4 chambers are formed by 102 h APF [12].

The adult female *Drosophila* possesses a pair of ovaries, each containing 16–20 ovarioles. Each ovariole consists of an apical germarium and a posterior series of six to seven consecutively developing egg chambers [16]. Each egg chamber consists of 15 nurse cells and one oocyte, and it develops through 14 morphologically distinct stages to reach the mature stage 14 egg chamber. Starting from stage 11, the nurse cells rapidly transfer their cytoplasmic contents to the oocyte through a process called nurse cell dumping, leaving behind the nurse cell nuclei and a small amount of cytoplasm [17]. By stage 13, the nurse cell nuclei begin to be eliminated. At the close of oogenesis at stage 14, all the nurse cell nuclei are cleared away, leaving only the fully developed oocyte [18]. Ultimately, a mature egg is formed, marking the completion of the oogenesis process in *D. melanogaster* [19].

*Drosophila* oogenesis involves the coordinated development of somatic and germ cells, with numerous signals playing essential roles in this process. Both the BMP and JAK/STAT pathways are required for regulation of the programmed proliferation and differentiation of PGCs [5]. BMP-pathway ligand Dpp is secreted by CCs. Only the PGCs in contact with CCs receive Dpp signaling, enabling them to develop into GSCs [20]. The JAK/STAT signaling pathway functions by regulating Dpp signaling [21]. *Stat92E* mutation reduces *Dpp* expression in CCs and ESCs. As a result, Dpp signaling is attenuated in GSCs adjacent to CCs, leading to the loss of repression on the pro-differentiation factor (*Bam*) and consequently causing premature differentiation of GSCs. A similar effect is observed with *Dpp* mutation [22]. Under *Stat92E* mutant conditions, overexpression of *Dpp* can still induce GSC over-proliferation, confirming that Dpp signaling acts downstream of JAK/STAT and that both pathways collectively influence GSC maintenance [23].

Sec61β is a subunit of the Sec61 complex, which functions as a conserved eukaryotic translocon responsible for translocation of nascent polypeptides into the endoplasmic reticulum (ER) [24,25,26]. As demonstrated in previous studies, *Sec61β* participates in autophagy suppression [27,28], directly interacts with microtubules, and localizes to the endomembrane system [29]. *Sec61β* is engaged in multiple biological processes, including ER homeostasis maintenance [28,29], cuticle protein secretion [30], and co-translational protein trafficking [25,31,32]. Depletion of *Sec61β* in mammalian cells and *Caenorhabditis elegans* induces mild ER stress [29]. In response to ER stress, cells activate a set of adaptive intracellular signal-transduction pathways, known as the unfolded-protein response (UPR) [33]. The UPR involves activation of three major canonical ER stress sensors—namely, inositol-requiring enzyme 1 (IRE1), protein kinase RNA-activated like ER kinase (PERK) and activating transcription factor 6 (ATF6). These ER stress transducers are localized to the ER membrane [34]. *Sec61β* is critical for *Drosophila* development, as loss of *Sec61β* results in embryonic lethality, though the underlying molecular mechanism remains elusive [29,30,35]. *Drosophila Sec61β* is homologous to human SEC61B. Dysfunction of SEC61B is closely linked to the pathological mechanisms of various diseases, forming a disease spectrum referred to as “Sec61 channelopathies”, such as autosomal dominant polycystic liver disease [36]. Abnormalities in SEC61B are also gaining attention in tumor biology [37].

According to modENCODE tissue expression data, *Sec61β* is highly expressed in both testes and ovaries of *Drosophila* (flybase) [38], implying that *Sec61β* might be critical for fly fertility. Our previous studies have shown that testis-specific knockdown of *Sec61β* results in smaller testes devoid of germ cells, eventually leading to male sterility [39]. However, the role of *Sec61β* in female fertility remains unclear. In this study, we found that knockdown of *Sec61β* in either germline cells or somatic cells of *Drosophila* ovary severely impairs female fertility and ovarian development. Knockdown of *Sec61β* in germ cells results in significantly smaller adult ovaries and a complete absence of germ cells in the germarium. This phenotype may involve the UPR pathway, which non-autonomously activates JAK/STAT signaling in adjacent somatic cells, thereby inhibiting GC differentiation while also autonomously disrupting cell-cycle progression during substantial GC proliferation, ultimately leading to the loss of GCs. Our findings reveal a previously unrecognized role for *Sec61β* in cell proliferation and differentiation that may be linked to “Sec61 channelopathies”, including tumorigenesis [37].

## 2. Results

### 2.1. Sec61β Is Essential for Female Fertility of D. melanogaster

To investigate the role of *Sec61β* in female reproduction, we employed two independent RNAi lines targeting distinct mRNA sequences of *Sec61β* to avoid possible off-target effects. qRT-PCR analyses confirmed that *Sec61β* expression levels in the ovaries were significantly reduced in flies expressing *Sec61β*-RNAi under the control of nosGal4 (nosGal4 > *Sec61β*-RNAi1; nosGal4 > *Sec61β*-RNAi2) compared to controls (nosGal4 > *w*^−^) (Figure 1B, *p* < 0.001). Importantly, knockdown of *Sec61β* in early germ cells severely impaired female fecundity, resulting in no egg laying by the females from either RNA1 line, while the control females laid nearly 486 ± 37 eggs within four days (Figure 1C). Overexpression of *Sec61β* rescued the female fertility defects caused by *Sec61β* knockdown (Figure 1C), which was accompanied by an elevated expression level of *Sec61β* in the rescue group (*nosGal4* > UAS-*Sec61β*; *Sec61β*-RNAi1) (Figure 1B). Since *Sec61β* is also abundantly expressed in ovarian somatic cells, we used tjGal4 to drive the knockdown of *Sec61β* in these cells. This similarly led to females failing to lay eggs (Figure 1C). These results indicate that *Sec61β* is indispensable for female fertility in both germ cells and somatic cells of the ovary. Depletion of *Sec61β* in either germ cells or somatic cells of the ovary results in complete sterility in females. As *Sec61β*-RNAi1 and *Sec61β-*RNAi2 had similar effects on female fertility, all subsequent experiments were conducted using *Sec61β*-RNAi1.

### 2.2. Sec61β Is Required for Oogenesis

**Figure 2:**
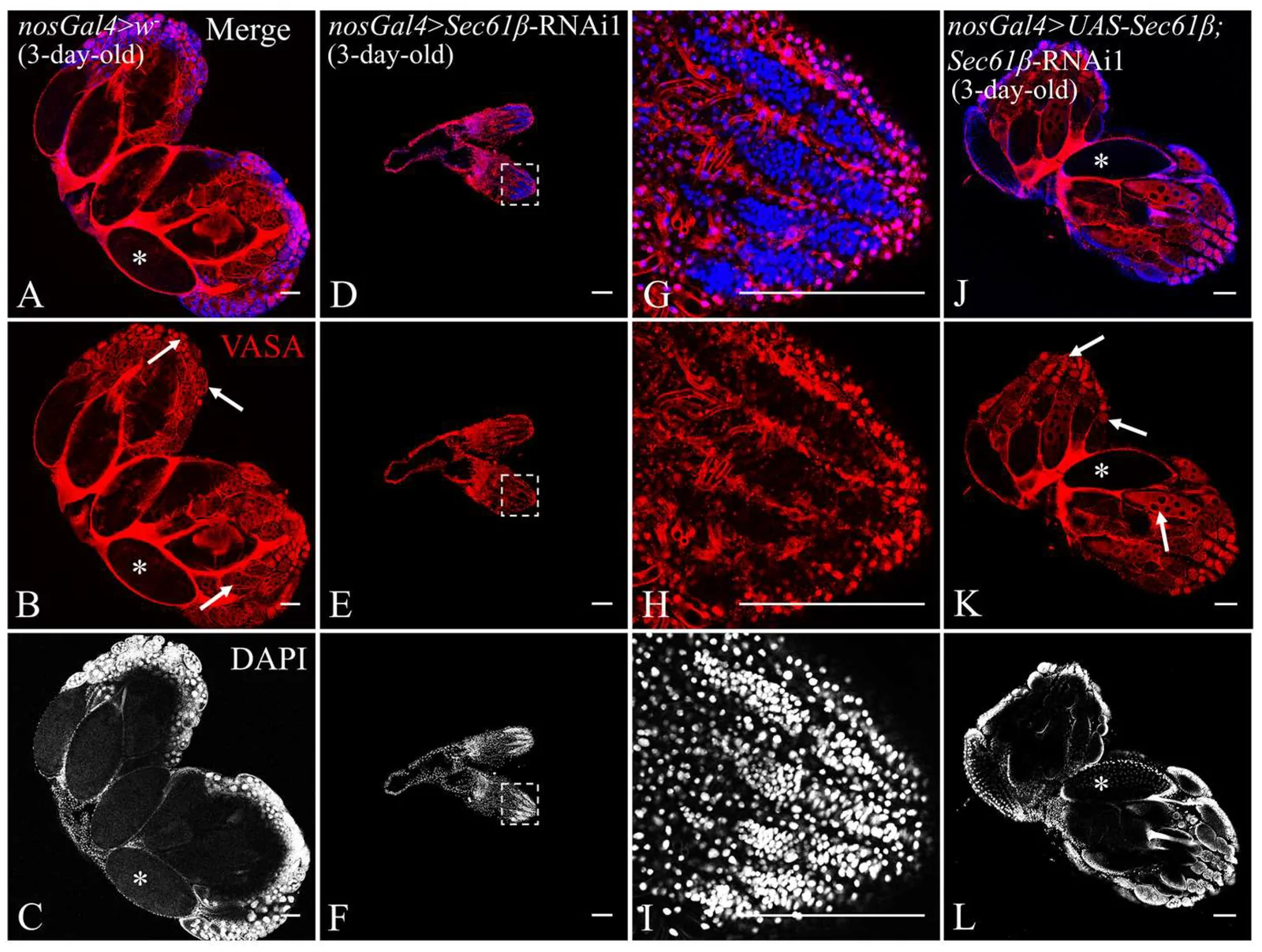
*Sec61β* knockdown results in severe defects in oogenesis and ovarian development in 3-day-old flies. (**A**–**C**) Overall view of control ovaries, showing germ cells at different developmental stages (arrows in (**B**)) and mature eggs (asterisks in (**A**–**C**)). (**D**–**I**) Gross appearance of *Sec61β* knockdown ovaries. (**G**–**I**) Anterior region of *Sec61β* knockdown ovaries, displaying the germarium (white dashed boxes in (**D**–**F**)). No germ cells are present within the ovarioles. (**J**–**L**) Gross appearance of rescue ovaries, showing germ cells at different developmental stages (arrows in (**K**)) and mature eggs (asterisks in (**J**–**L**)). Anti-VASA antibody (red) labels germ cells; DAPI (blue and white) labels cell nuclei. Scale bars: 100 μm.

**Figure 3:**
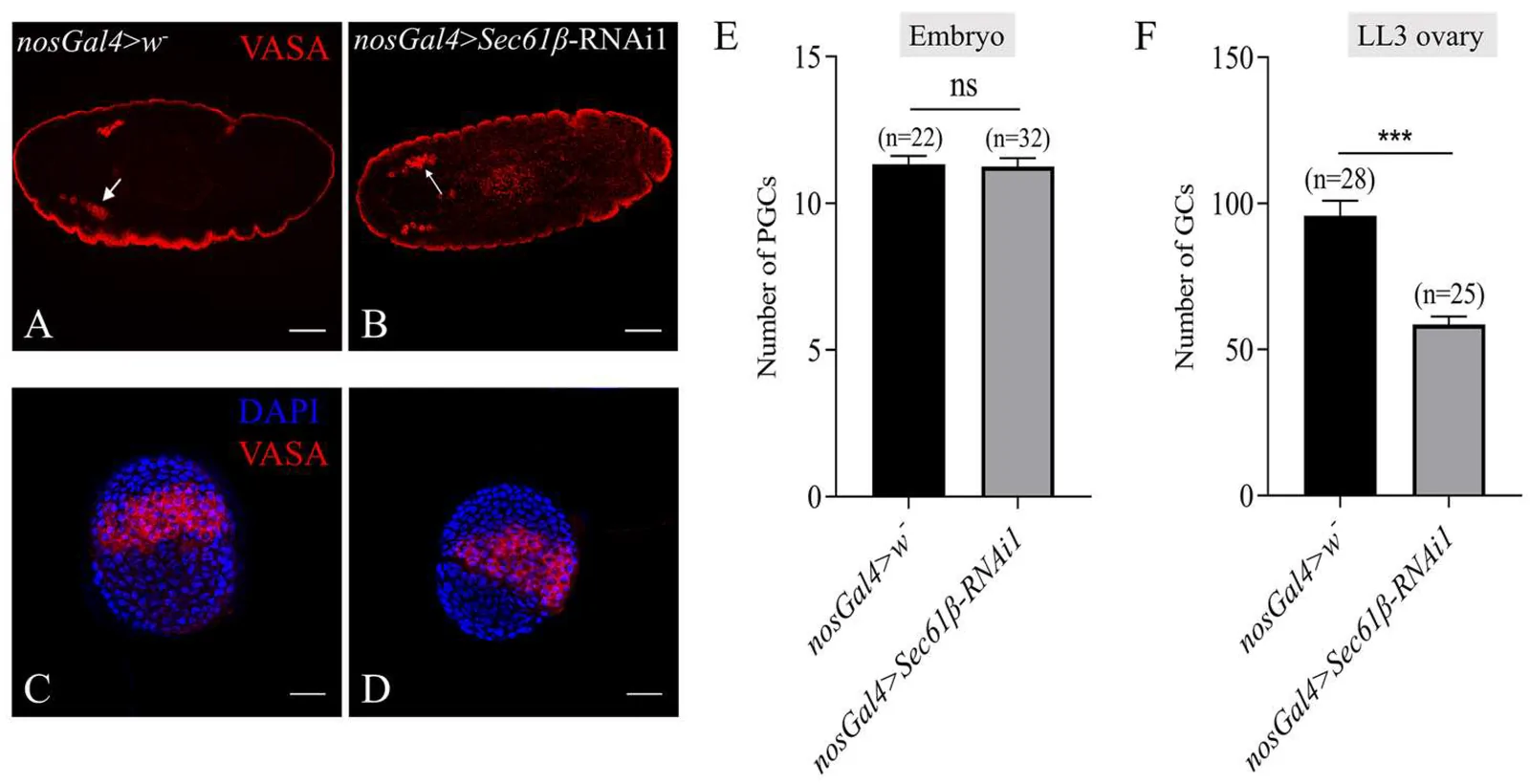
*Sec61β* knockdown leads to a reduction in germ cell (GC) number within ovaries at the late-third-instar larval (LL3) stage. (**A**) Control embryo and (**B**) *Sec61β* knockdown embryo around 10–11 h post egg-laying. Primordial germ cells (PGCs, arrows in (**A**,**B**)) are present in the posterior regions of both control and *Sec61β* knockdown embryos. (**C**,**D**) Overall view of the control (**C**) and *Sec61β* knockdown (**D**) LL3 ovary. *Sec61β* knockdown led to a significant reduction in GC numbers compared to control LL3 ovaries. Anti-VASA antibody (red) labels PGCs and GCs. DAPI (blue) labels cell nuclei. Scale bars: 75 μm (**A**,**B**); 25 μm (**C**,**D**). (**E**) Quantification of PGCs in a single primordial gonad from control and *Sec61β* knockdown embryos. n: number of embryos counted. (**F**) Quantification of GCs in control and *Sec61β* knockdown LL3 ovaries. n: number of ovaries counted. ns: non-significant, *** *p* < 0.001.

To investigate the potential mechanism by which *Sec61β* knockdown impairs female fertility, we dissected adult ovaries and examined oogenesis via immunostaining, using an anti-VASA antibody to label germ cells and DAPI to counterstain nuclei. We found that the both 1d (Figure 1D–I) and 3d (Figure 2A–C) control ovaries were well-developed and filled with clearly visible germ cells at different stages (arrows in Figure 1H and Figure 2B). The ovarioles contained apical germarium (red square bracket in Figure 1I), egg chambers at various developmental stages (green square bracket in Figure 1I), and mature eggs (asterisks in Figure 1D–F and Figure 2A–C). In contrast, *Sec61β* knockdown resulted in atrophied ovaries, with ovarioles appearing collapsed and empty. No discernible germarium or egg chambers were observed, and oocytes were completely absent (Figure 1J–O). Phalloidin staining revealed that although the ovarioles were atrophied, they remained discernible as discrete structures (Figure S2). The defects caused by *Sec61β* knockdown did not diminish with increasing age, as the ovaries of 3d *Sec61β* knockdown females remained apparently smaller than controls (Figure 2D–I) and lacked germ cells (Figure 2D–F). Conversely, the ovarian development in the rescue group (*nosGal4* > UAS-*Sec61β*; *Sec61β*-RNAi1) was nearly indistinguishable from that of the controls, with visible egg chambers at various stages and the presence of mature eggs (Figure 2J–L). These results indicate that *Sec61β* is essential for oogenesis during the adult stage.

### 2.3. Sec61β Knockdown Results in Loss of Germ Cells in LL3 Ovaries

As *Sec61β* knockdown in the early germline led to a complete loss of germ cells in adult females, we wondered at which development stage the germ cell loss occurred. During normal embryonic development, PGCs initially form at the posterior pole of the embryo. They are subsequently internalized with the endoderm and gradually migrate to a pair of developing gonads, which are located in the posterior region of the embryo [40]. Here, we found that in both control and *Sec61β* knockdown embryos, the PGCs indicated by Vasa signals were migrating toward the posterior gonads (arrows in Figure 3A,B), and each migrating germ cell stream contained approximately 12 PGCs (Figure 3E), indicating that *Sec61β* knockdown does not affect PGC formation or migration during embryonic development.

Next, we dissected ovaries from late-third-instar larvae (LL3). The *Sec61β* knockdown LL3 ovaries appeared slightly smaller (Figure 3D) than those of the control group (Figure 3C). Importantly, although germ cells (GCs) were present in the ovaries of the *Sec61β* knockdown group, their numbers were significantly lower than those observed in the control group (Figure 3F). We then examined TF development in LL3 ovaries. Immunofluorescence staining results revealed that evenly spaced stacks of TFs, each consisting of 8–10 TFCs, were observed in both control (Figure S3A,B) and *Sec61β* knockdown (Figure S3C,D) LL3 ovaries. Furthermore, TFs were observed in both the pupal (Figure S3E–H) and adult ovaries (Figure S3I–P) of the *Sec61β* knockdown group, comparable to those in control groups. These results suggest that *Sec61β* knockdown leads to a reduction in the number of GCs in the third-instar larval stage, which is not associated with defects in niche establishment.

**Figure 4:**
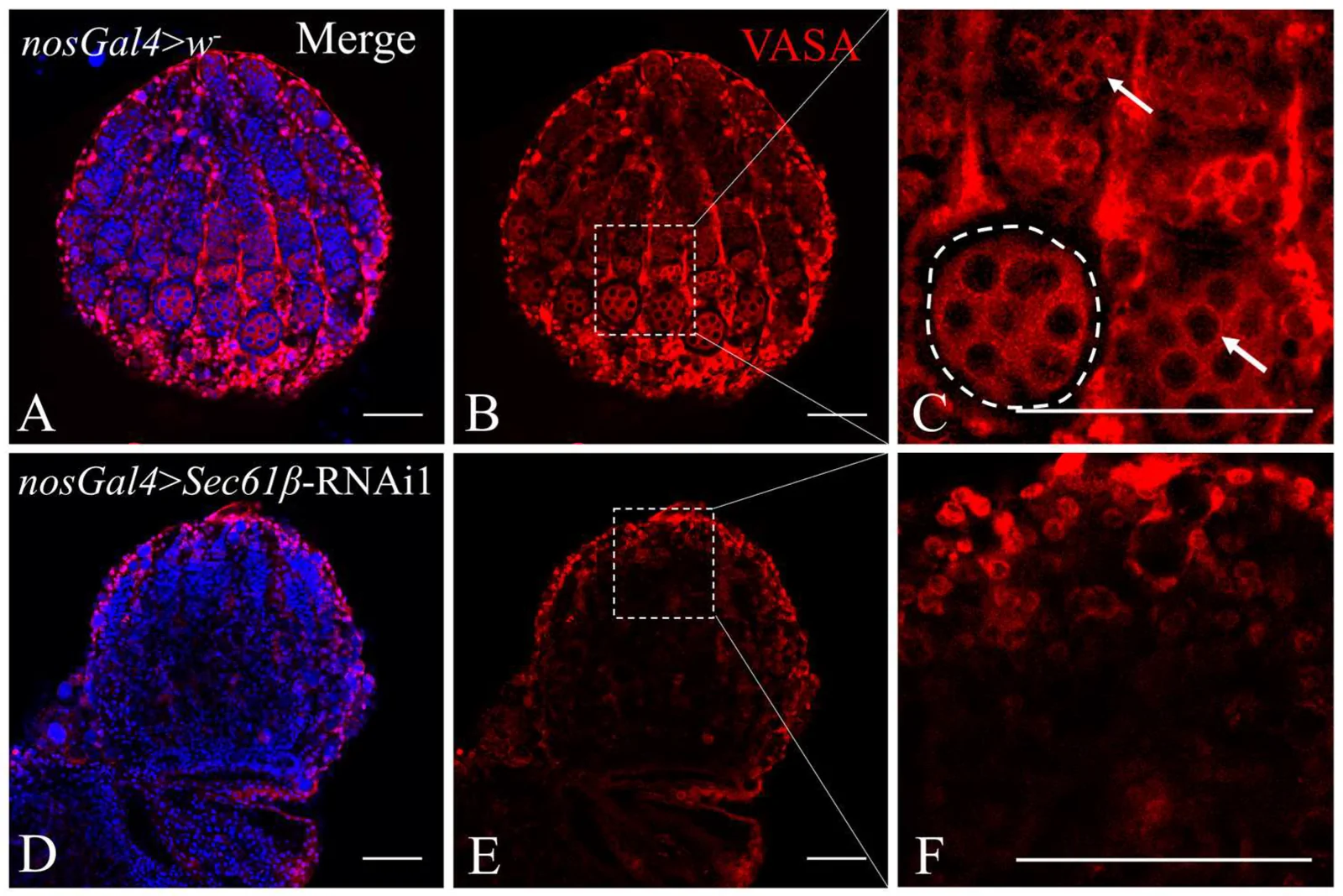
*Sec61β* knockdown results in the complete loss of germ cells (GCs) in pupal ovaries. (**A**,**B**) Overall view of control pupal ovaries. (**D**,**E**) Gross appearance of *Sec61β* knockdown pupal ovaries. *Sec61β* knockdown led to smaller pupal ovaries compared to the control. (**C**,**F**) Ovarioles from the control (**C**) and *Sec61β* knockdown (**F**) pupal ovaries. The control ovarioles contain cystoblast cysts at various development stages (arrows in (**C**)) and have developed egg chambers (dashed circles in (**C**)). In contrast, *Sec61β* knockdown ovarioles (**F**) exhibit a complete absence of GCs. Anti-VASA antibody (red) labels GCs. DAPI (blue) labels nuclei. Scale bars: 50 μm (**A**,**B**,**D**,**E**) and 70 μm (**C**,**F**).

To further track the developmental status of GCs, we dissected and examined the pupal ovaries. Immunofluorescence staining with anti-VASA antibody and DAPI revealed that control pupal ovaries (Figure 4A–C) had developed egg chambers at various stages, along with GCs inside (Figure 4C). In contrast, *Sec61β* knockdown led to a complete absence of GCs in pupal ovaries (Figure 4D–F). Consequently, no egg chambers were present (Figure 4F). These results indicate that the oogenesis defects caused by *Sec61β* knockdown initiates at the LL3 stage and progress until the pupal stage, when all GCs are completely lost.

### 2.4. Sec61β and Ocnus (ocn) Do Not Mutually Rescue Female Fertility

**Figure 5:**
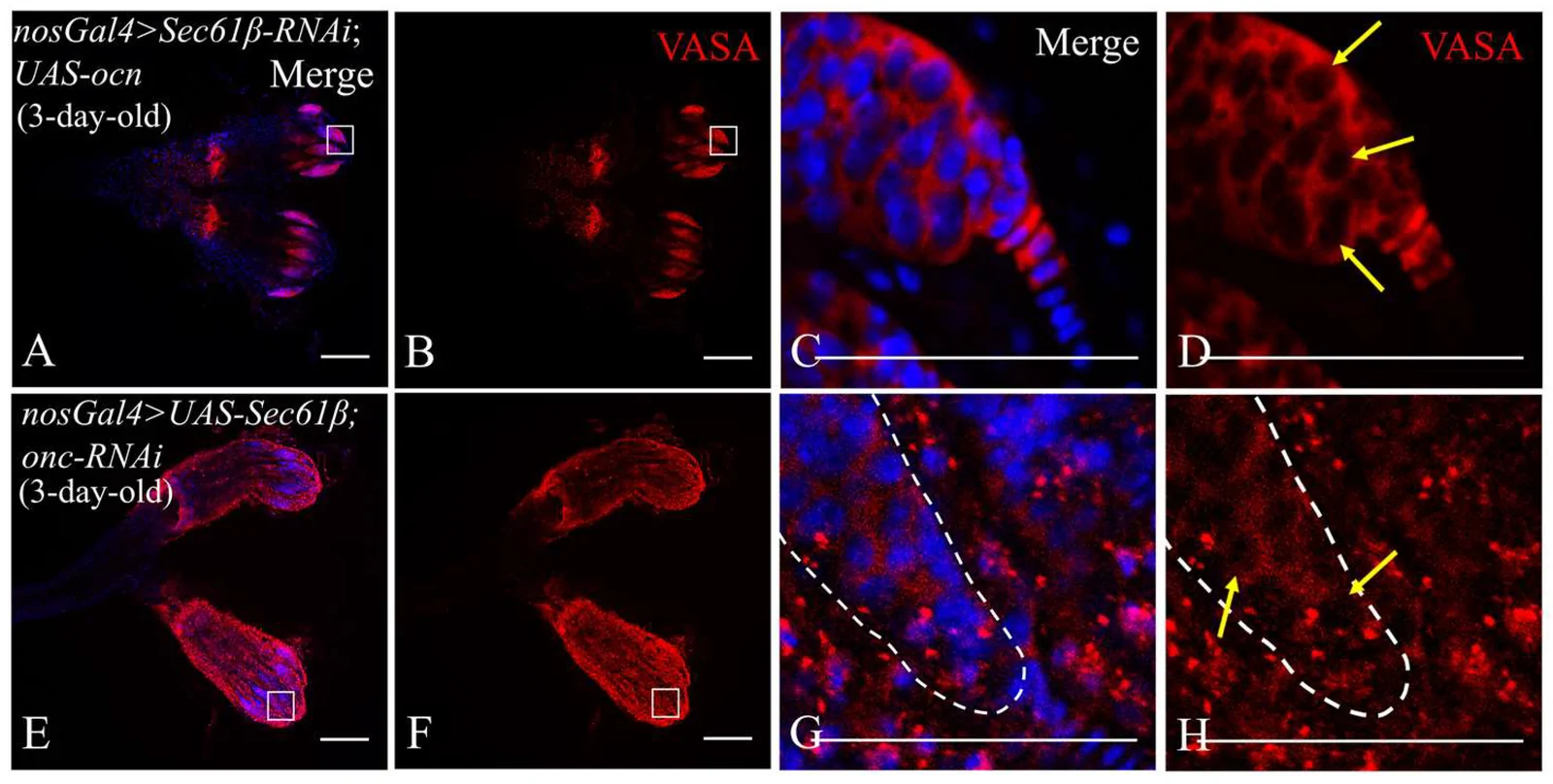
The relationship between *Sec61β* and *ocn* during ovarian development in *D. melanogaster*. (**A**–**D**) Ovaries from 3d adult flies with *ocn* overexpression under the background of *Sec61β* knockdown. The solid-line boxes indicate the magnified anterior ovariole regions (**A**,**B**). *Ocn* overexpression partially rescued the complete loss of germ cells caused by *Sec61β* knockdown with the appearance of germ cells (GCs) (arrows in (**D**)). (**E**–**H**) Ovaries from adult flies with *Sec61β* overexpression under the background of *ocn* knockdown. The solid-line boxes indicate the magnified anterior ovariole regions (**E**,**F**). *Sec61β* overexpression leads to slightly discernible ovarioles (dashed lines in (**G**,**H**)) and the presence of faint Vasa-positive signals in the germarium (arrows in (**H**)). Anti-VASA antibody (red) labels GCs. DAPI (blue) labels cell nuclei. Scale bars: 100 μm (**A**,**B**,**E**,**F**) and 40 μm (**C**,**D**,**G**,**H**).

*ocn* encodes a protein highly expressed in the *Drosophila* testis. We previously showed that *ocn* depletion in the testis causes germ cell loss and infertility and that *ocn* overexpression partially rescues *Sec61β* knockdown-induced male sterility but not vice versa [39]. This prompted us to test whether a similar interaction exists in the female germline. In contrast to the testis, *ocn* overexpression in female germ cells failed to rescue female infertility resulting from *Sec61β* knockdown. Similarly, *Sec61β* overexpression was also unable to rescue the female infertility induced by *ocn* knockdown (Table S2), consistent with observations in males [39]. Notably, although *ocn* overexpression did not restore female fertility, it partially rescued the complete loss of GCs in ovarioles caused by *Sec61β* knockdown in adult females (arrows in Figure 5D). However, egg chambers still failed to develop, and no mature eggs were produced (Figure 5A,B). Likewise, *Sec61β* overexpression partially rescued the GC loss resulting from *ocn* knockdown (arrows in Figure 5H), albeit to a lesser extent than the rescue effect of *ocn* overexpression on the *Sec61β* knockdown background (Figure 5E–H). This suggests that *Sec61β* and *ocn* are functionally associated during oogenesis; however, this interaction is not essential for this process in *D. melanogaster*.

### 2.5. Sec61β Knockdown Leads to UPR and Autophagy in GCs

**Figure 6:**
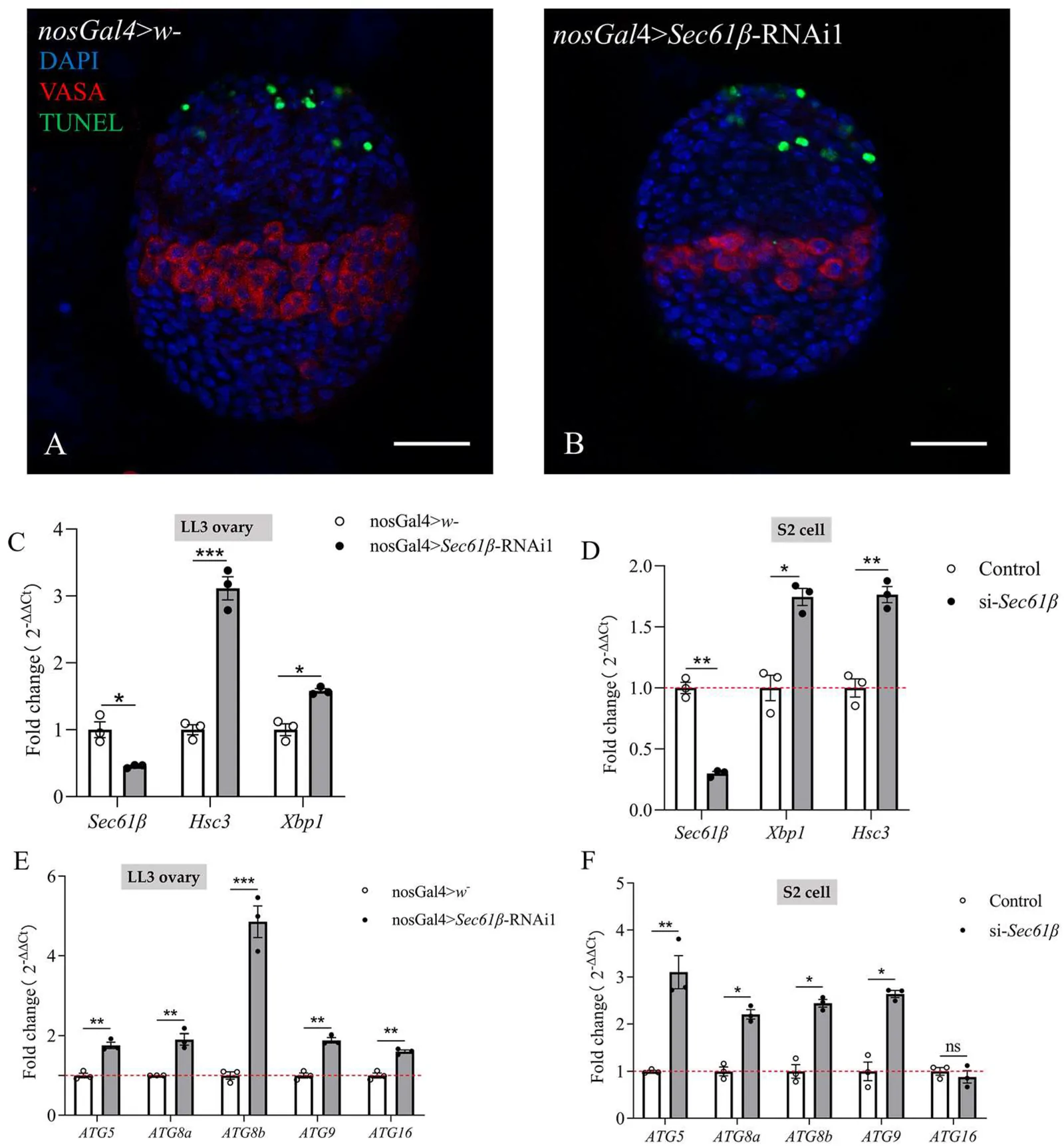
*Sec61β* knockdown induces the unfolded protein response (UPR). (**A**) Control and (**B**) *Sec61β* knockdown LL3 ovaries show no apoptosis in GCs. Anti-VASA antibody (red) labels GCs. TUNEL (green) labels apoptotic cells. DAPI (blue) labels nuclei. Scale bars: 25 μm. (**C**) qRT-PCR analysis of the effect of *Sec61β* knockdown on expression levels of UPR-related genes *Xbp1* and *Hsc3* in LL3 ovaries. (**D**) qRT-PCR analysis of the effect of *Sec61β* knockdown in S2 cells on expression levels of UPR-related genes *Xbp1* and *Hsc3*. (**E**) qRT-PCR analysis of the effect of *Sec61β* knockdown on expression levels of autophagy-related genes in LL3 ovaries. (**F**) qRT-PCR analysis of the effect of *Sec61β* knockdown in S2 cells on expression levels of autophagy-related genes. The red dotted lines in (**D**–**F**) correspond to the “1” on the vertical axis. n: number of embryos counted, * *p* < 0.05, ** *p* < 0.01, *** *p* < 0.001.

Depletion of *Sec61β* leads to ER stress, which, in turn, induces apoptosis [29,41]. To determine whether germ cell loss induced by *Sec61β* knockdown occurs via apoptosis, we performed TUNEL staining on LL3 ovaries. In both control and knockdown flies, apoptotic signals were restricted to anterior cells (ACs) (Figure 6A,B), with no detectable apoptosis in germ cells. This suggests that TUNEL-positive ACs likely function in the physiological clearance of excess somatic cells in the ovary, which has been demonstrated previously [42], and that the reduction in the number of germ cells induced by *Sec61β* knockdown is not mediated by apoptosis at this stage.

Recent studies have shown that *Sec61β* deficiency disrupts the functional connection between the translocon complex and IRE1, thereby triggering the UPR [29]. Previous research indicates that when *Drosophila* is exposed to ER stress that activates the UPR, the level of IRE1-dependent spliced *Xbp1* mRNA increases, and *Hsc3* transcripts are also elevated [43]. We sought to determine whether *Sec61β* knockdown induces the UPR in fly ovaries and therefore examined the transcript levels of the spliced forms of *Xbp1* and *Hsc3*. qRT-PCR results showed that the mRNA levels of both spliced *Xbp1* and *Hsc3* were significantly increased (Figure 6C). This indicates that *Sec61β* knockdown activates the UPR.

A known consequence of UPR activation is the degradation of misfolded/unfolded proteins via autophagy [44]. Consequently, we examined how autophagy-related genes change following *Sec61β* knockdown. The results showed significant upregulation of *ATG5*, *ATG8a*, *ATG8b*, *ATG9*, and *ATG16* (Figure 6E). To avoid the complexity of ovarian cell composition of fly ovaries, we knocked down *Sec61β* using siRNA in *Drosophila* S2 cells and detected the expression of related genes. qRT-PCR results confirmed that *Sec61β* knockdown indeed induces the upregulation of UPR and autophagy-related genes (Figure 6D,F).

### 2.6. Sec61β Knockdown May Affect JAK/STAT Signaling

**Figure 7:**
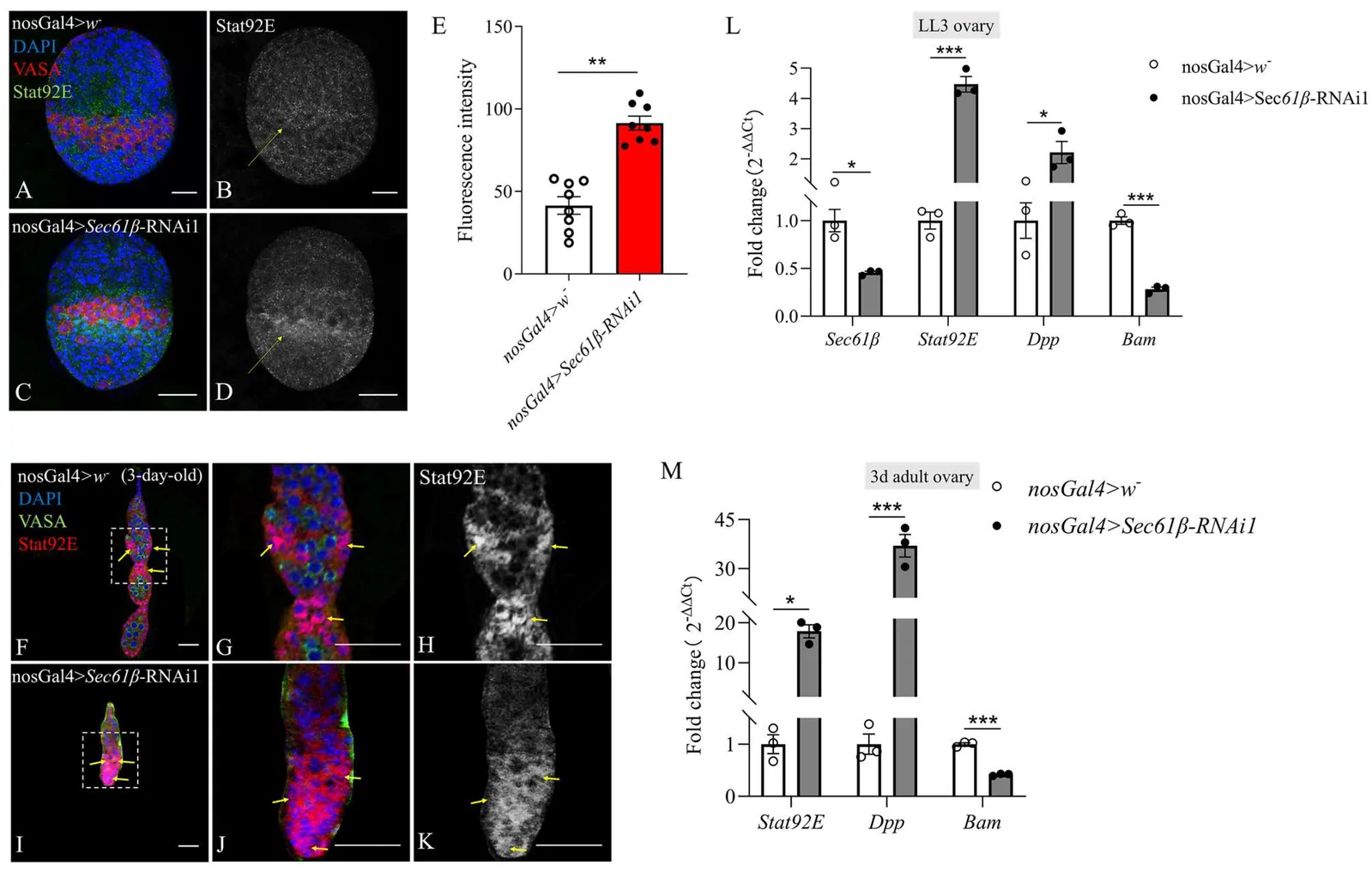
*Sec61β* knockdown induces hyperactivation of the JAK/STAT signaling pathway in the ovaries of *D. melanogaster*. (**A**,**B**) Control and (**C**,**D**) *Sec61β* knockdown LL3 ovaries. Stat92E is predominantly expressed in the intermingled cells (ICs) surrounding the GCs (arrow in (**B**)), and its expression is elevated upon *Sec61β* knockdown (arrow in (**D**)). Anti-VASA antibody (red) labels GCs. Anti-Stat92E antibody (green) labels Stat92E. DAPI (blue and white) labels cell nuclei. Scale bars: 20 μm. (**E**) Fluorescence intensity of Stat92E in control and *Sec61β* knockdown LL3 ovaries. ** *p* < 0.01. (**F**–**H**) Immunostaining analysis of ovarioles from control and (**I**–**K**) *Sec61β* knockdown adult ovaries. In the control group, Stat92E is primarily distributed in follicle stem cells (FSCs) and follicle cells (FCs) (arrows in (**F**–**H**)). In contrast, Stat92E is highly accumulated in numerous somatic cells at the tip of the ovarioles in the *Sec61β* knockdown flies (arrows in (**I**–**K**)). G and J are magnified views of the white dashed boxes in (**F**,**I**). Anti-VASA antibody (green) labels GCs. Anti-Stat92E antibody (red) labels Stat92E. DAPI (blue and white) labels nuclei. Scale bar: 20 μm. (**L**) qRT-PCR analysis of *Stat92E*, *Dpp* and *Bam* expression levels in the LL3 ovaries following *Sec61β* knockdown. (**M**) qRT-PCR analysis of *Stat92E*, *Dpp* and *Bam* expression levels in 3-day-old adult ovaries following *Sec61β* knockdown. * *p* < 0.05, ** *p* < 0.01, *** *p* < 0.001.

In the *Drosophila* intestinal epithelium, ER stress in neighboring cells, such as enterocytes, has been shown to non-autonomously activate PERK via JAK/STAT signaling in intestinal stem cells, promoting their proliferation [45]. Therefore, we asked whether knockdown of *Sec61β* influencing ovarian development is involved in STAT signaling. To test this, we used Anti-Stat92E antibody to stain LL3 ovaries. This revealed that *Sec61β* knockdown significantly increased Stat92E expression in the intermingled cells (ICs) surrounding GCs (arrows in Figure 7B,D).

As escort cells (ECs), follicle stem cells (FSCs), and most follicle cells (FCs) derive from ICs interspersed with germline cells [46], we also examined adult ovaries and found that in control ovarioles, Stat92E was predominantly localized to FSCs and FCs (arrows in Figure 7F–H). *Sec61β* knockdown led to the absence of germ cells, while numerous somatic cells accumulated within the ovarioles, where Stat92E was highly accumulated (arrows in Figure 7I–K).

qRT-PCR analyses also showed that *Sec61β* knockdown resulted in a significant increase in *Stat92E* transcription levels in LL3 ovaries (Figure 7L) and 3-day-old adult ovaries (Figure 7M). Since *Stat92E* can enhance the expression of its target *Dpp*, which, in turn, suppresses *Bam* expression to regulate germline stem-cell self-renewal and prevent differentiation [47], we tested the expression of *Dpp* and *Bam* in *Sec61β* knockdown ovaries. *Dpp* was significantly upregulated, while *Bam* was significantly downregulated (Figure 7L,M). These molecular changes are indicative of hyperactivation of JAK/STAT signaling, which may cause germ cells to be blocked in an undifferentiated state and unable to proceed through regular differentiation, ultimately leading to their loss by the pupal stage.

### 2.7. Sec61β Knockdown Causes Defects in Cell-Cycle Progression

**Figure 8:**
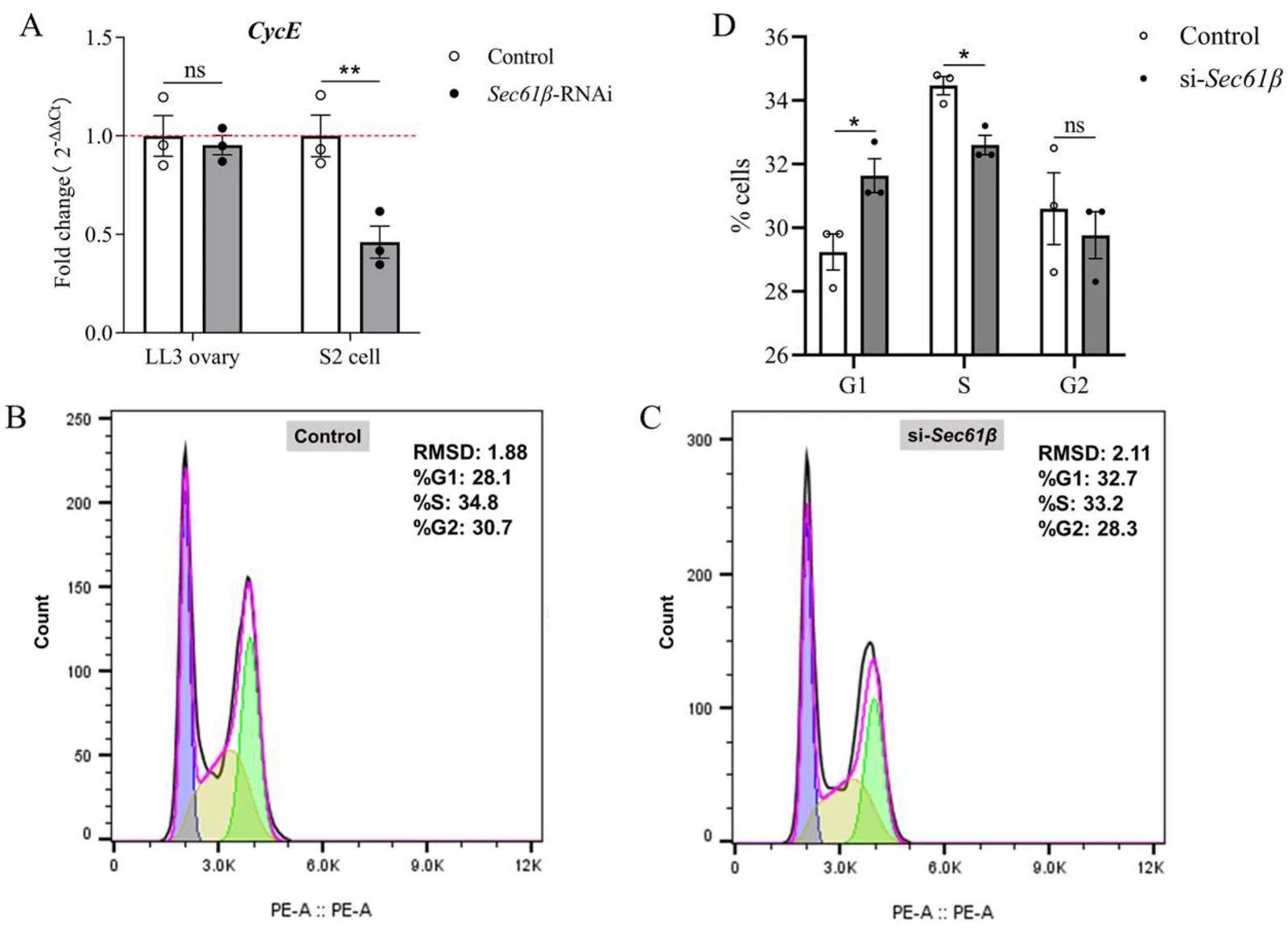
*Sec61β* knockdown causes defects in cell-cycle progression. (**A**) qRT-PCR analysis of the effect of *Sec61β* knockdown on expression levels of *CycE* in LL3 ovaries and S2 cells. The red dotted lines correspond to the “1” on the vertical axis. (**B**) PE-A histogram showing the cell-cycle profile of the control group. (**C**) PE-A histogram showing the cell-cycle profile of the *Sec61β* knockdown group. (**D**) Statistical analysis was performed on the proportions of G1-, S-, and G2-phase cells between the control and knockdown groups. *Sec61β* knockdown led to a significant increase in the proportion of G1-phase cells, a significant decrease in the proportion of S-phase cells, and a decreasing trend in the proportion of G2-phase cells. ns: non-significant, * *p* < 0.05, ** *p* < 0.01.

Since both the UPR and autophagy can delay cell-cycle progression, affecting the transition from the G1 to G2 phase [48,49,50], we examined the expression of *CycE*, a key gene for the G1/S transition in mitosis [51]. Although *CycE* showed no change in LL3 ovaries, its transcript level was significantly reduced in S2 cells upon *Sec61β* knockdown (Figure 8A). Using flow cytometry analysis, we detected significant changes in the proportions of G1- and S-phase cells in *Sec61β* knockdown S2 cells. The knockdown group exhibited a significantly increased proportion of G1-phase cells and a significantly decreased proportion of S-phase cells compared to the control group, while the proportion of G2-phase cells also showed a decreasing trend (Figure 8B–D). This is consistent with the qRT-PCR results (Figure 8A), which showed that *Sec61β* knockdown leads to a significant reduction in *CycE* in S2 cells, thereby blocking subsequent cell-cycle events in a subset of cells. Based on the staining results of adult ovaries, which revealed a significant increase in somatic cells upon *Sec61β* knockdown, the change in *CycE* in LL3 ovaries might be masked by somatic cell proliferation. In summary, these results suggest that *Sec61β* knockdown may be insufficient to induce apoptosis but is sufficient to activate certain adaptive UPR signaling pathways, enabling ovarian GCs to overcome the effects of ER stress caused by *Sec61β* knockdown.

## 3. Discussion

In this study, we identified a previously unrecognized role of *Sec61β* during ovarian development and oogenesis in *D. melanogaster*. We show that knockdown of *Sec61β* in either the early germ cells (GCs) or the somatic cells of the ovary results in complete female sterility, which is attributable to a catastrophic failure of oogenesis. Depletion of *Sec61β* in early germ cells leads to loss of GCs commencing at the larval stage; this effect is not due to apoptosis but may be mediated through the induction of endoplasmic reticulum (ER) stress and autophagy, which leads to hyperactivation of the JAK/STAT signaling in adjacent somatic cells and consequently inhibits germ cell differentiation. ER stress in germ cells also induces defects in cell-cycle progression, thereby suppressing PGC proliferation at the late third-instar larval stage (LL3). These findings suggest that *Sec61β* plays an indispensable role in maintaining female germline development, likely by regulating proteostasis, signal transduction, and cell-cycle progression.

We observed here that *Sec61β* knockdown in early germ line cells did not impair the formation and migration of PGCs during embryogenesis but led to a significant reduction in GC number in LL3 ovaries, with a complete loss of GCs and apparently smaller ovaries by the pupal stage. This may reflect the fundamental differences in protein synthesis and secretion requirements between early and late oogenesis. During embryonic and early larval stages, PGCs are formed that are mainly under maternal control and primarily exhibit a state of slow proliferation. The stem cell niche determining their future fate has not yet been formed; therefore, the requirement for *Sec61β* is not urgent. By mid-third instar, the stem cell niche forms, and stem cells establish, rapidly proliferate and differentiate [6]. This series of process requires rapid synthesis and transport of large amounts of secretory/membrane proteins (such as various signaling molecules, receptors, adhesion molecules, etc.). Therefore, at this time point, the absence of *Sec61β* results in catastrophic consequences, including ER stress; dysregulation of signaling pathways; and, ultimately, loss of germ cells. Numerous studies have shown that reduced larval ovary size and loss of GCs are closely associated with the establishment of the stem cell niche. For instance, mutations in *Drosha* or *Pasha* led to a decrease in the number of terminal filaments (TFs) and terminal filament cells (TFCs) per TF, as well as defective TF morphogenesis in larval ovaries, consequently resulting in a significant reduction in GC number and smaller ovary size [5]. Ovaries with *Lola* knockdown exhibit a reduced number of TFs and disorganized structure, manifesting as smaller larval ovaries [11]. However, our study found that *Sec61β* knockdown in early germ cells does not affect the assembly of TFs, further supporting its function in processing and transporting secretory and membrane proteins required for the rapid proliferation and differentiation of germ cells at this stage. Previous studies have found that in adult *Drosophila* ovaries, upon the loss of GSCs, TFCs can persist for up to 3 weeks, and CCs can continue to exist for up to 18 days. Inner-germarium sheath cells (IGSs) and FSCs sequentially enter the vacated niche. However, IGSs are less stable and completely disappear 8–9 days after GSC loss, ultimately leaving only FSCs in the original germarium. Moreover, ectopic FSCs are capable of responding to major niche signals and undergo substantial proliferation upon receiving Dpp signaling [52]. Therefore, although *Sec61β* knockdown leads to the complete absence of GCs in the ovarioles of adult flies, a large number of somatic cells, most likely FSCs, remain.

We previously demonstrated that Sec61β can directly interact with Ocn, probably at the nuclear envelope, in S2 cells and that overexpression of *ocn* partially restores fertility in *Sec61β* knockdown males in vivo [39]. In the current study, we found that overexpression of *ocn* failed to rescue female fertility in the *Sec61β* knockdown background, although overexpression of *ocn* promoted oogenesis in *Sec61β* knockdown ovaries and vice versa; however, germ cells ultimately failed to develop into mature eggs. This result is expected, given that *ocn* exhibits a male-specific expression pattern but *Sec61β* is highly expressed in both testes and ovaries of *Drosophila* (flybase) [38]. This result indicates that *Sec61β* and *ocn* do not play completely overlapping roles during spermatogenesis and oogenesis.

Newly synthesized proteins are modified and folded within the ER. If misfolded proteins accumulate in the ER, they induce ER stress and activate the unfolded protein response (UPR). Sec61β, an essential component of the ER translocon, is critical for the maintenance of ER homeostasis, and its depletion has been demonstrated to induce ER stress in both mammalian cells and *C. elegans* [29]. The UPR is a conserved signaling network responsible for monitoring and adjusting ER function to maintain ER homeostasis [48]. It consists of three signaling arms: IRE1, PERK, and ATF6 [53], which coordinate adaptive responses to alleviate ER stress, including upregulation of ER chaperones and attenuation of global protein translation. Specifically, activated IRE1 splices *Xbp1* mRNA to generate a transcription factor that induces chaperone expression; PERK phosphorylates eIF2α to reduce translation initiation, thereby lowering the protein-folding burden on the ER; and ATF6 is cleaved to a soluble transcription factor that also contributes to the cellular response to ER stress. However, if ER homeostasis cannot be restored, prolonged UPR signaling can trigger programmed cell death [54]. In our study, both *Hsc3* (an ER chaperone) and *Xbp1* were significantly upregulated in *Sec61β* knockdown LL3 ovaries, indicating that ER stress is indeed induced under these conditions. Notably, despite the clear induction of UPR markers, we did not detect evidence of apoptosis in germ cells by TUNEL staining. This suggests that the ER stress triggered by *Sec61β* knockdown is sufficient to activate the adaptive UPR but does not reach the threshold required to initiate apoptotic cell death.

Increased ER stress in neighboring cells has been demonstrated to non-autonomously activate PERK through JAK/STAT signaling in intestinal stem cells to induce their proliferation [45]. A similar association was observed in the ovaries of *D. melanogaster*. *Sec61β* knockdown in GCs resulted in a significant increase in the protein level of Stat92E in somatic cells adjacent to GCs in LL3 ovaries, and enrichment of Stat92E was also observed in somatic cells within adult ovarioles. Overexpression of the JAK/STAT pathway kinase Hop in ICs has been reported to lead to a significant increase in the number of ICs, which inhibit PGC proliferation [55]. This constitutes a feedback mechanism that achieves homeostasis and coordinated growth between somatic cells and the germline in the larval ovary [3]. Furthermore, stimulation of JAK/STAT signaling in somatic cells induced by *Sec61β* knockdown resulted in marked upregulation of its downstream gene, *Dpp*, and significant downregulation of *Bam*, which inhibits GC differentiation in the LL3 ovary. These findings suggest that *Sec61β* in the germline may act as a key regulator coordinating the intercellular signaling circuit that balances GSC self-renewal versus differentiation.

In addition to UPR activation, we observed a robust induction of autophagy in both LL3 ovaries and *Drosophila* S2 cells following *Sec61β* knockdown, as evidenced by significant upregulation of autophagy-related genes *ATG5*, *ATG8a*, *ATG8b*, *ATG9*, and *ATG16*. Importantly, both ER stress and excessive or prolonged autophagy have been shown to impair cell-cycle progression, leading to G1- or G2-phase arrest [48,49,50,56]. To test whether *Sec61β* depletion similarly affects the cell cycle, we turned to S2 cells, which allow for clean separation of cell autonomous effects from complex tissue interactions. In *Sec61β* knockdown S2 cells, we observed a marked decrease in *CycE* transcript levels and, by flow cytometry, a clear G1-phase arrest (increased G1 fraction with concomitant reduction in the S phase). These results provide direct cell-level evidence that *Sec61β* depletion impairs G1/S progression. Notably, the *CycE* transcript level was not significantly altered in whole LL3 ovary extracts. One possible explanation for this discrepancy is that *CycE* expression in whole-ovary extracts reflects contributions from both germline and somatic cells. Because somatic cells continue to proliferate during larval development, while germ cells are the primary population affected by *Sec61β* depletion, subtle changes in germ-cell-specific *CycE* expression may be masked by somatic cell signals. Under physiological conditions, PGCs proliferate to approximately 100–120 cells during the LL3 [5]. Our finding that *Sec61β* knockdown induces G1/S arrest in S2 cells, combined with the in vivo observation of reduced germ cell numbers in LL3 ovaries, strongly suggests that a similar cell-cycle defect contributes to the failure of germ cell expansion in vivo. Future experiments are required to directly verify cell-cycle arrest in the germline compartment and to establish its causal relationship with the UPR and autophagy.

In addition to its germline-autonomous function, *Sec61β* is required in ovarian somatic cells, as depletion of *Sec61β* using tjGal4 resulted in the absence of recognizable ovaries in adult females. Although the developmental onset and cellular basis of this severe phenotype remain to be determined, a plausible explanation is that *Sec61β* is required for the establishment and/or maintenance of ovarian somatic cell lineages during ovarian development. In particular, follicle stem cell progenitors (FSCPs) generated during larval development give rise to adult follicle stem cells and follicle cells, which are essential for the encapsulation of germline cysts and the formation of individual egg chambers. Developmental ablation of these progenitors not only causes severe egg-chamber formation defects and gross ovarian distortion but also induces cell death in both germline and somatic cells [57]. Therefore, *Sec61β* depletion may impair the development and survival of follicle cell progenitors and their descendants, thereby compromising germline cyst encapsulation and secondarily undermining germ cell maintenance.

## 4. Materials and Methods

### 4.1. Fly Stocks

The nosGal4 line was kindly provided by Professor Zhaohui Wang at the Institute of Genetics and Developmental Biology, Chinese Academy of Sciences (CAS). The tjGal4 line was kindly provided by Professor Lei Zhang at the Shanghai Institute of Biochemistry and Cell Biology, CAS, Shanghai, China. Transgenic fly line *Sec61β* RNAi1 (*Sec61β*-RNAi1, TH201500823.S) and *Sec61β* RNAi2 (*Sec61β*-RNAi2, BS50626) were obtained from the Tsinghua Fly Center (Beijing, China) and the Bloomington Drosophila Stock Center (BDSC, Bloomington, IN, USA), respectively. *Sec61β*-RNAi1 targets the gene region from position 278 to 298, while *Sec61β*-RNAi2 interferes with the gene region from position 854 to 874. The overexpression line (UAS-*Sec61β*) was from BDSC.

To achieve early germline-specific RNAi or overexpression, we used the nosGal4 driver. Virgin females of nosGal4 were crossed with either *Sec61β*-RNAi1 or *Sec61β-*RNAi2 males to generate *Sec61β* knockdown flies (*nosGal4* > *Sec61β*-RNAi1 or *nosGal4* > *Sec61β*-RNAi2). Similarly, virgin females of nosGal4 were crossed with UAS-*Sec61β* males to obtain *Sec61β* overexpression flies (*nosGal4* > UAS-*Sec61β*). The *Drosophila* strains with both overexpression of *Sec61β* and knockdown of *ocn* (UAS-*Sec61β*; *ocn*-RNAi) and those with overexpression of *ocn* and knockdown of *Sec61β* (*Sec61β*-RNAi; UAS-*ocn*) were all constructed and maintained by our laboratory. The strains (UAS-*Sec61β*; *Sec61β*-RNAi) with both *Sec61β* RNAi and overexpression elements were generated by a sequence of crossing. The detailed protocols are shown in Figure S1. The male flies (UAS-*Sec61β*; *Sec61β*-RNAi1) were arranged to cross with nosGal4 virgin females to obtain *Sec61β* rescue flies (*nosGal4* > UAS-*Sec61β*; *Sec61β*-RNAi1). Flies from the crosses of nosGal4 females and wild-type males (*w*^1118^, nosGal4 > *w*^−^) were used as the corresponding controls.

All flies were reared on a standard cornmeal/yeast medium at 25 °C with a humidity of approximate 75% and a photoperiod of 12L:12D (light:dark).

### 4.2. Female Fertility Test

For each biological replicate, 3- to 5-day-old (3–5 d) virgin females (*n* = 10) were allowed to mate with 1-day-old (1d) *w*^1118^ males (*n* = 15) for approximately 12–16 h. After mating, the *w*^1118^ males were removed, and the mated females were allowed to lay eggs for 4 days. The eggs were collected, counted, and incubated at 25 °C under 75% humidity for about 30 h. Hatching rates were calculated by dividing the number of hatched eggs by the total egg count [58]. At least three independent trials per cross type were conducted.

### 4.3. qRT-PCR

Total RNA was successfully extracted using the Trizol protocol (Invitrogen, Waltham, MA, USA), ensuring efficient isolation of high-quality RNA. The cDNA synthesis process employed the EasyScript First-Strand cDNA synthesis SuperMix Kit (TransGen Biotech, Beijing, China) in accordance with the manufacturer’s guidelines, utilizing two micrograms of RNA per reaction to guarantee optimal yield and quality of synthesized cDNA. For qRT-PCR, we utilized a Miniopticon system (Bio-Rad, Hercules, CA, USA) with Platinum SYBR Green qPCR SuperMix reagent (TransGen Biotech, Beijing, China), chosen for its high efficiency in amplification. Specific primers were designed based on sequences retrieved from FlyBase (https://flybase.org/), with detailed primer information provided in Table S1. The PCR reaction comprised an initial denaturation step at 95 °C for 3 min, followed by 40 amplification cycles: denaturation at 95 °C for 10 s, annealing at 58 °C for 30 s, and extension at 72 °C for 20 s. A melting curve was generated post amplification by incrementally increasing the temperature from 55 °C to 98 °C in 1 °C increments, which was crucial for verifying product specificity. Gene expression levels were normalized relative to the *rp49* reference gene using the 2^−ΔΔCT^ method. ΔΔCt = (Ct*_taget_* − Ct*_rp49_*) Treated − (Ct*_taget_* − Ct*_rp49_*) Control. All experiments were conducted with three biological replicates and two technical replicates for each biological replicate.

### 4.4. Immunofluorescence Staining and TUNEL Assays

Embryos were collected 0.5 h after oviposition and aged for 17 h at 18 °C to reach stage ES 13 or 19.5 h at 18 °C to reach stage ES 14 [40]. To prepare the embryos for imaging, they were exposed to a 50% hydrogen peroxide solution (30%, Sigma-Aldrich (St. Louis, MO, USA)) for 2–3 min to soften the chorion. Subsequently, the embryos were washed in embryo wash buffer (0.7% NaCl, 0.05% Triton X-100 (BioFroxx, Einhausen, Germany)) and subjected to dechorionation by vigorous shaking in a 1:1 mixture of heptane and methanol for 30 s. Following dechorionation, the embryos were washed three times with methanol and stored at −20 °C until further use [59].

Ovaries at different developmental stages were dissected in phosphate buffer solution (PBS) and immediately fixed in 4% paraformaldehyde for 30 min at room temperature. Following fixation, the samples underwent three consecutive washes in PBST (PBS with 0.1% Triton X-100), each lasting 10 min. The tissues were then blocked for 30 min at room temperature in a blocking solution containing 5% normal goat serum to reduce non-specific binding. Subsequently, the samples were incubated overnight at 4 °C with primary antibody diluted in PBST. After this step, the tissues underwent three additional washes in PBST, each lasting 15 min. The secondary antibody was added and allowed to bind for 2 h at room temperature in the dark. Following another set of three 10-min washes in PBST, the samples were sealed and examined using a Leica SP8 laser confocal microscope (Leica Microsystems GmbH, Wetzlar, Germany) to obtain fluorescence images.

The primary antibodies were used at the following dilutions: rat anti-Vasa antibody (1:50; Developmental Studies Hybridoma Bank, Iowa, IA, USA, AB760351) [60,61], rabbit anti-Stat92E (1:5000; from Professor Zhaohui Wang at the Institute of Genetics and Development Biology, Chinese Academy of Sciences, Beijing, China) [62,63] and mouse anti-En antibody (1:20, clone 4D9, Developmental Studies Hybridoma Bank, Iowa, IA, USA) [11]. Secondary antibodies were applied at the following concentrations: rat 594 (1:200; Abbkine, Wuhan, China, A23420), rabbit 488 (1:200, Abbkine, Wuhan, China, A23220), mouse 488 (1:200; Abbkine, Wuhan, China, A23210), rabbit 594 (1:500, Abbkine, Wuhan, China, A23420) and Phalloidin (1:200, Abbkine, Wuhan, China, BMD00084). A TUNEL assay was performed using the In Situ Cell Death Detection Kit (Roche, Mannheim, Germany) [64,65], following the manufacturer’s instructions. All samples were mounted on glass slides using a 4′-6-diamidino-2-phenylindole (DAPI) solution at 2 μg ml^−1^ (Solarbio, Beijing, China) for nuclear staining.

### 4.5. S2 Cell Culture and Transfection

*Drosophila* S2 cells were generously provided by Professor Xi Zhou at the Wuhan Institute of Virology, CAS. S2 cells were cultured in Schneider’s *Drosophila* medium (Gibco, Waltham, MA, USA) supplemented with 10% fetal bovine serum (Every Green, Zhejiang Tianhang Biotechnology Co., Ltd., Huzhou, Zhejiang, China) and 0.01% penicillin–streptomycin (Gibco, Waltham, MA, USA) at 27 °C. Every 3–4 days, the S2 cells were passaged into new plates at a 1:3 ratio. Before transfection, S2 cells were seeded in 12-well plates until they reached 70–80% confluence per well [61]. To knock down *Sec61β* in S2 cells, siRNA targeting *Sec61β* (siRNA-*Sec61β*) was designed and synthesized by Wuhan AuGCT Biotechnology Co., Ltd. (Wuhan, China). The specific sequences of the siRNA are listed in Table S1. Transfection of S2 cells with siRNA was performed using Lipofectamine 2000 Transfection Reagent (Invitrogen, Thermo Fisher Scientific, Waltham, MA, USA) according to the manufacturer’s instructions. The transfection procedure involved mixing 2.4 μL of Lipo2000 with 125 μL of Schneider’s *Drosophila* medium in one tube and 5 μL of siRNA with 125 μL of Schneider’s *Drosophila* medium in another tube, followed by incubation at room temperature for 5 min. The two mixtures were then combined and incubated at room temperature for an additional 15–20 min before being added to the S2 cells. Transfected cells were harvested 48 h post transfection for RNA extraction and qRT-PCR analysis. Three biological replicates were performed for this experiment.

### 4.6. Quantitative Analysis of PGC Differentiation

PGCs were identified by positive VASA staining. The number of PGCs in embryonic and third-instar larvae was manually counted [5].

### 4.7. Flow Cytometry Analysis

S2 cells were collected and centrifuged at 1000× *g* for 5 min to pellet the cells. The supernatant was aspirated, and approximately 1 mL of pre-chilled PBS was added to resuspend the cells. The cells were pelleted again by centrifugation, and the supernatant was removed. The EP tube was gently tapped to disperse the cells appropriately and avoid clumping. While vortexing, 1 mL of pre-chilled 75% ethanol was added, and the cells were gently pipetted to mix. The cells were fixed overnight at −20 °C. After fixation, the cells were centrifuged at 1000× *g* for 5 min, the supernatant was aspirated, and 1 mL of pre-chilled PBS was added to resuspend the cells. The cells were centrifuged again, and the supernatant was removed. Propidium iodide (PI) staining solution was prepared according to the protocol of the Cell Cycle and Apoptosis Kit (SEVEN Biotech, Beijing, China). Then, 500 μL of PI staining solution was added to each cell sample, and the cell pellet was slowly and fully resuspended. The samples were incubated for 15 min at room temperature in the dark. Cell clumps were removed by filtering the cell samples through a cell strainer. Cell-cycle distribution was analyzed using a flow cytometer. DNA content analysis was performed using FlowJo software (version 10.9).

### 4.8. Statistical Analysis

Results are presented as means ± standard error (SE) with a sample size of n ≥ 3. Statistical analyses were performed using GraphPad Prism version 9.0. For comparisons among multiple developmental stages, data were analyzed by one-way analysis of variance (ANOVA). If ANOVA indicated significant differences, further pairwise comparisons were performed using a two-tailed Student’s *t*-test. When evaluating differences between specific means, a two-tailed Student’s *t*-test was employed. Statistical significance was defined as *p* < 0.05 (*), *p* < 0.01 (**), *p* < 0.001 (***).

## 5. Conclusions

In conclusion, our study demonstrates that *Sec61β* is essential for the massive proliferation of primordial germ cells (PGCs) during *Drosophila* oogenesis. As an essential component of the ER translocon, knockdown of *Sec61β* in early germ cells triggers the UPR and autophagy, which non-autonomously induces hyperactivation of the JAK/STAT pathway in adjacent somatic cells, thereby inhibiting GCs differentiation. Concurrently, the UPR and autophagy in germ cells affect the transition from the G1 to S phase, thereby inhibiting the substantial proliferation of GCs during the late-third-instar larval stage. These two mechanisms collectively lead to the complete loss of germ cells in the pupal and adult stages, ultimately resulting in female sterility. Future studies will focus on the specific impact of *Sec61β* knockdown on the cell cycle and whether it causes global translation inhibition within GCs.

## Data Availability

The original contributions presented in this study are included in the article/Supplementary Material. Further inquiries can be directed to the corresponding author.

## Supplementary Materials

The following supporting information can be downloaded at: https://www.mdpi.com/article/10.3390/ijms27177640/s1.

## Abbreviations

The following abbreviations are used in this manuscript:

PGC	Primordial Germ Cell
TFC	Terminal Filament Cell
CC	Cap Cell
IC	Intermingled Cell
GSC	Germline Stem Cell
LL3	Late Third-instar Larva
TF	Terminal Filament
APF	After Puparium Formation
CB	Cystoblast
GC	Germ Cell
EC	Escort Cell
FC	Follicle Cell
FSC	Follicle Stem Cell
AC	Anterior Cell
ER	Endoplasmic Reticulum
UPR	Unfolded Protein Response
IGS	Inner-Germarium Sheath cell
