# Supplementary material for: Sec61β Deficiency Disrupts Drosophila Oogenesis Through UPR-Mediated Defects in Germ Cell Proliferation and Differentiation
Source: Int J Mol Sci. 2026 Aug 26;27(17):7640. doi: 10.3390/ijms27177640 (PMC13566306; doi:10.3390/ijms27177640)
Supplement: Supplementary file 1 [file ijms-27-07640-s001.zip › Supplementary figures and tables.pdf]

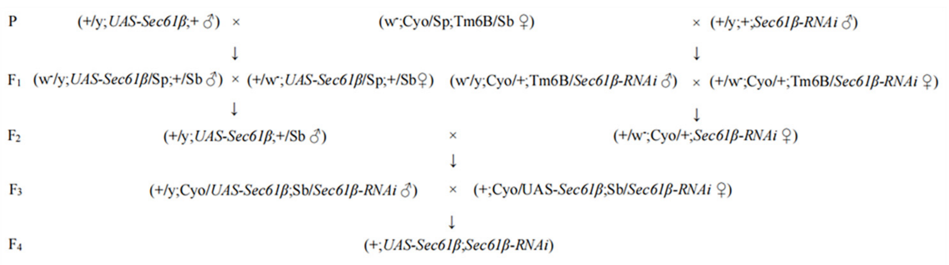

Figure S1. The diagram of the construction process for generating the rescue fly line UAS-*Sec61β*; *Sec61β*-RNAi.

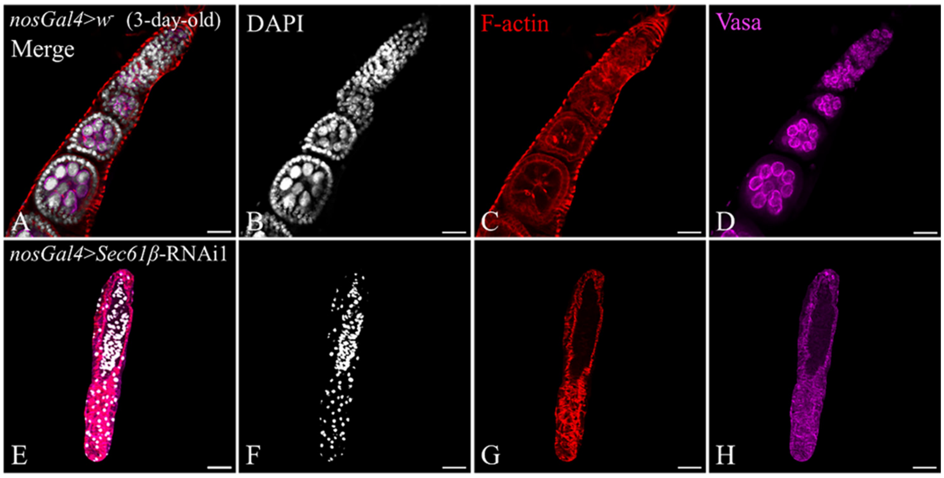

Figure S2. *Sec61β* knockdown does not affect the independence of ovarioles. **(A-D)** Immunostaining analysis of ovarioles from control and **(E-H)** *Sec61β* knockdown adult ovaries. Although the *Sec61β* knockdown ovarioles are shriveled, they maintain their independence. Phalloidin (red) labels the F-actin cytoskeleton, anti-Vasa antibody (magenta) labels germ cells, and DAPI (white) labels cell nuclei. Scale bar: 25 μm.

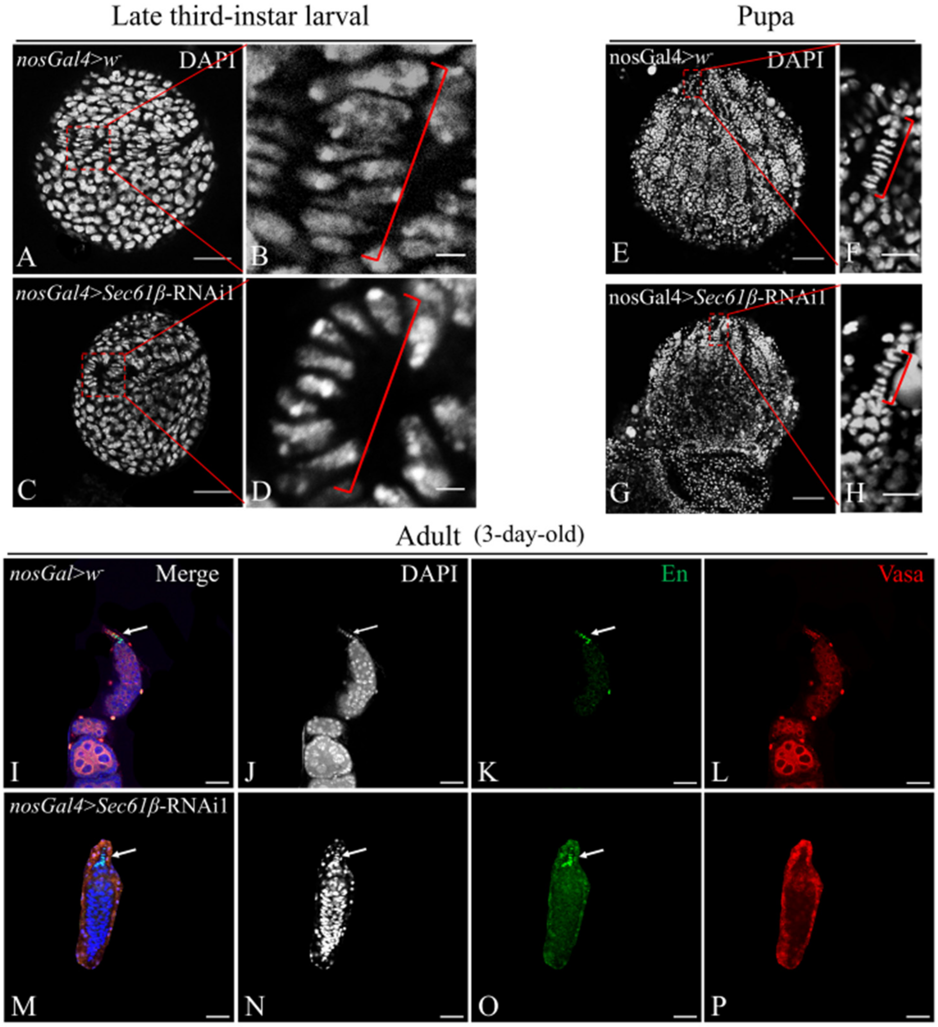

Figure S3. *Sec61β* knockdown does not affect terminal filament (TF) formation in the niche. (**A-D**) TF structure in third-instar larval stage, (**E-H**) pupal stage, and (**I-P**) 3-day-old adult stage ovaries of control and knockdown groups. In both control (A, B, E, F, I-L) and knockdown (**C, D, G, H, M-P**) ovaries, the TFs within the niche consist of 8-10 terminal filament cells (red brackets in **B, D, F, H**) arranged in a regular pattern. Anti-VASA antibody (red) labels germ cells, anti-En antibody (green) labels terminal filament cells, and DAPI (white) labels nuclei. The terminal filament cells exhibit a flattened ellipsoid shape. Scale bars, 25 μm (**A, C, I-P**); 50 μm (**E, G**); 3 μm (**B, D**); 5 μm (**F, H**).

Table S1. Primers used in this study.

Gene name	Sequence (5'-3')
Rp49	F: CGGTTACGGATCGAACAAGC<br>R: CTTGCGCTTCTTGGAGGAGA
Sec61β	F: AAATTGTCGGCGCCACGTA<br>R: CGTGTAGAAACGCCACATGC
Stat92E	F: CAGCGTATGGCGGCTTACTA<br>R: TCTTCTCGGTGACCAACTGC
Dpp	F: AGGACAAGCTGAAGCCAGAC<br>R: AGAGCTTCTTCATCGGCTCG
Bam	F: TGAGTTCCTCTCCGCTCTGA<br>R: GATGATGGCACTTCCTCGGT
Hsc3	F: TGGGAGGTGAGGACTTCGAT<br>R: CGCCCTCGAAGAAGGATTCA
Xbp1	F: GAACAGCCACAATCACAGCG<br>R: ATCACGTCCGTTGAGTCGTC
ATG5	F: ATATGCTTCCAGGCGGATCG<br>R: AACCACACAGCTCCATCCTG
ATG8a	F: CGTCGCAAATATCCAGACCG<br>R: GAAGAAGAGGGCATCCTCGG
ATG8b	F: CAAGCGTATCAATCTGCGTCC<br>R: TAGTCCTTGTCGAAGTGCTCC
ATG9	F: AGCAGAAGCACGGATTCACA<br>R: GCAGTGCATCACAAAGGCAA
ATG16	F: AAATCGCAAGGAGTGCGACA<br>R: CTTCAGGTTGTCCGCCTTCA
CycE	F: GCCCAATCGCTATCCTTCCA<br>R: AGCACATCTCGTCGGTTCTG
Sec61β siRNA	Sence: AAUUUCAUGUGGAAACACACCTT<br>Antisence: GGUGUGUUUCCACAUGAAAUUTT

Table S2. Female fertility test.

Cross group(♀×♂)	Egg counted	***p*-value**
(1) *nosGal4*> w^{-}×w^{1118}	486.33±33.14	
(2) *nosGal4*>*Sec61β*-RNAi; UAS-*ocn*× w^{1118}	0	(2) vs. (1)<br>p<0.001
(3) *nosGal4*>UAS-*Sec61β*; *ocn*-RNAi× w^{1118}	0	(3) vs. (1)<br>p<0.001
